# Online food delivery systems: barriers to achieving public health nutrition in the Philippines

**DOI:** 10.1017/S1368980023000782

**Published:** 2023-06

**Authors:** Dalmacito Austria Cordero

**Affiliations:** Department of Theology and Religious Education (DTRE), De La Salle University, 1004 Taft Avenue, Manila, Philippines

**Keywords:** Online food delivery, Philippines, Nutrition, Public Health

I read with great interest a recent article where the authors interestingly highlighted the potential of online food delivery (OFD) systems in improving public health nutrition. These OFD systems like online school canteens became popular during the COVID-19 pandemic in Australia, and they were helpful in different ways: they can provide access to large and increasing proportions of the child population; they are more amenable to government regulation or mandatory policy to regulate the availability and nutritional quality of foods; their labeling can be an effective strategy in modifying dietary habits during critical periods in child development, etc^(1)^. I fully support these observations, however, as mentioned by the authors; further research is necessary to determine certain barriers in using OFD systems that can hinder in achieving the goal of public health nutrition. This is what I am going to highlight, especially in developing countries like the Philippines.

The food service industry was one of the essential services that were allowed to operate during the pandemic in the Philippines. The OFD statistics in the Philippines showed that there were 2·9 million users of online delivery platforms, and the largest segment is in the restaurant-to-consumer delivery service, which logged 9·3 million users. The food delivery market is projected to reach an annual revenue of 333·30 million US dollars in 2022^(2)^. The two most popular OFD platforms are Foodpanda and GrabFood, while other restaurants/stores have their own OFD systems and riders such as McDonald’s, Jollibee, Shakey’s Pizza and many others. Throughout the pandemic period, these OFD stores/restaurants played a significant role in the provision of all types of food to the public, not only to fill the hungry stomachs of those who were stuck in their homes due to quarantines/lockdowns but also those that contribute to unhealthy eating habits.

It is important to determine some barriers concerning OFD systems that do not help in achieving public health nutrition. First, there are instances that some of the online stores deliver the ordered food without proper preparation or packaging which can harm consumers. Due to large volumes of orders, there is a tendency to overcook, undercook and inappropriately prepare orders. What is more unfortunate is that some are already spoiled due to poor packaging before reaching the customer making them unsafe for consumption. The primary purpose of packaging is to protect the food from contamination, maintain the right food temperatures, and prevent it from spilling and spoiling^(3)^. These scenarios result in increased risk of lower than expected nutritional quality and higher risk of food poisoning. Second, some OFD systems and restaurants present attractive marketing practices which could potentially lead to vulnerable people like children. There is an array of ultra-processed foods and drinks that are readily available at low cost like salty snacks, carbonated beverages and away-from-home meals^(4)^. The consumption of ultra-processed foods has been associated in recent prospective studies with increased risks of all-cause mortality and chronic diseases such as cancer, CVD, hypertension and dyslipidemia^(5)^. Lastly, it is interesting to note that one-third of all meals consumed by Filipinos were ordered from restaurants, while two-thirds were home-cooked. Fifty per cent of customers decide what they want to eat based on what they see on the mobile app and on what will satisfy their cravings for that specific food^(6)^. This means that the unhealthy eating habits of some Filipinos will continue because it became a part of their culture to ‘click’ on their mobile app to satisfy their food cravings. With this, OFD systems should strictly follow the government policies and guidelines regarding safe practices of food preparation and delivery. There is a dire need for food safety training for OFD workers and certification concerning acceptable procedures to eradicate incidences of foodborne disease outbreaks^(7)^.

Public health nutrition involves programs and policies that promote optimal nutrition and the well-being of the general public. The immediate provision of food services during a pandemic is a must but needs to be carefully planned and properly carried out. The different marketing strategies in OFD systems are acceptable for economic growth, but it is important to consider that they exist not simply for profit but to contribute in nation building – a healthy society, especially during health crises.
